# How Behavior Change Strategies are Used to Design Digital Interventions to Improve Medication Adherence and Blood Pressure Among Patients With Hypertension: Systematic Review

**DOI:** 10.2196/17201

**Published:** 2020-04-09

**Authors:** Kobra Etminani, Arianna Tao Engström, Carina Göransson, Anita Sant’Anna, Sławomir Nowaczyk

**Affiliations:** 1 Center for Applied Intelligent Systems Research Halmstad University Halmstad Sweden; 2 Center for Research on Welfare, Health and Sport Halmstad University Halmstad Sweden

**Keywords:** digital intervention, hypertension, medication adherence, behavior change, intervention mapping, matrix of change objective.

## Abstract

**Background:**

Information on how behavior change strategies have been used to design digital interventions (DIs) to improve blood pressure (BP) control or medication adherence (MA) for patients with hypertension is currently limited.

**Objective:**

Hypertension is a major modifiable risk factor for cardiovascular diseases and can be controlled with appropriate medication. Many interventions that target MA to improve BP are increasingly using modern digital technologies. This systematic review was conducted to discover how DIs have been designed to improve MA and BP control among patients with hypertension in the recent 10 years. Results were mapped into a matrix of change objectives using the Intervention Mapping framework to guide future development of technologies to improve MA and BP control.

**Methods:**

We included all the studies regarding DI development to improve MA or BP control for patients with hypertension published in PubMed from 2008 to 2018. All the DI components were mapped into a matrix of change objectives using the Intervention Mapping technique by eliciting the key determinant factors (from patient and health care team and system levels) and targeted patient behaviors.

**Results:**

The analysis included 54 eligible studies. The determinants were considered at two levels: patient and health care team and system. The most commonly described determinants at the patient level were lack of education, lack of self-awareness, lack of self-efficacy, and forgetfulness. Clinical inertia and an inadequate health workforce were the most commonly targeted determinants at the health care team and system level. Taking medication, interactive patient-provider communication, self-measurement, and lifestyle management were the most cited patient behaviors at both levels. Most of the DIs did not include support from peers or family members, despite its reported effectiveness and the rate of social media penetration.

**Conclusions:**

This review highlights the need to design a multifaceted DI that can be personalized according to patient behavior(s) that need to be changed to overcome the key determinant(s) of low adherence to medication or uncontrolled BP among patients with hypertension, considering different levels including patient and healthcare team and system involvement.

## Introduction

### Overview

There is increasing demand for the wide adoption of digital tools and interventions as the entire healthcare ecosystem struggles to deal with the biggest burden of the 21st century: chronic diseases. However, both theory and practice on how to create digital interventions (DIs) remain underdeveloped and under analyzed. Therefore, we performed a systematic literature review to identify the specific components previously used in DIs targeting medication adherence (MA) and/or blood pressure (BP) control in patients with hypertension. We present the results of this analysis within a matrix of change objectives (MoCO) using a framework called Intervention Mapping (IM). This framework allowed us to identify both the activities that are common across different interventions and the goals these activities were intended to achieve. The ultimate impact of health innovations depends on how research evidence and existing theories are used [1]. Thus, organizing the published studies in a framework that considers an existing common theory supports more effective intervention development in the future. At the same time, outlining existing research in such a map provides more profound insight into the strengths, focus, and weaknesses of DIs that have already been developed.

### Background

Hypertension, or high BP, is a global public health issue. Approximately 40% of people worldwide are estimated to have hypertension [2]. According to the World Health Organization (WHO) Global Brief on Hypertension [2], 9.4 million deaths annually are attributed to complications of hypertension. Hypertension was identified as the third most important factor for disability-adjusted life years globally in 2002 [3] and as the leading risk factor for global burden of disease in 2010 [4]. Based on the Framingham Heart Study [5], a recent article estimated the direct cost associated with hypertension to be 51.3 million EUR across five European countries [6]. Hypertension is a major modifiable risk factor for cardiovascular diseases [4]. To put it in a different way, patients with controlled BP are 50% less likely to suffer a cardiovascular event compared to those with uncontrolled BP [7].

### Medication Adherence and Blood Pressure Control

The WHO defines adherence to long-term therapy as “the extent to which a person's behavior—taking medication, following a diet, and/or executing lifestyle changes—corresponds to agreed recommendations from a health care provider” [8]. In this work, we focus specifically on adherence to a medication regimen to improve BP control.

Studies have repeatedly found that long-term adherence to hypertensive medication is low. A recent systematic review concluded that over 45% of hypertensive patients failed to comply with their medication regimen [9]. It has been estimated that increasing the adherence rate to antihypertensive therapy to 70% would reduce cardiovascular events by over 80,000 cases across the five European countries listed in the article based on the Framingham Heart Study [6].

Adherence to medications is a behavioral process driven by the interaction of many factors, which the WHO classifies into 5 categories: socioeconomic factors, factors associated with the health care team and system in place, disease-related factors, therapy-related factors, and patient-related factors [8]. Several patient-related factors, including lack of understanding of their disease, lack of involvement in the treatment decision-making process, and suboptimal medical literacy, contribute to medication nonadherence [10]. Horne and Weinman correlated MA with two main factors: patients' personal beliefs about the necessity of their prescribed medication and their concerns about taking it [11]. They measured these two factors using the Beliefs about Medicines Questionnaire and showed, in a later systematic review of 94 studies, that higher MA was consistently associated with stronger perceptions of necessity and weaker perceptions of concern [12].

### Behavior Change Theories

A meta-analysis reviewing MA intervention for adults with hypertension concluded that the most promising intervention components were those linking adherence behavior with habits, giving adherence feedback to patients, self-monitoring of BP, using pill boxes and other special packaging, and motivational interviewing [13]. Although the literature lists many factors correlated with MA, the causal relationships with such factors must be explored. Behavioral psychology professionals have developed several theories that try to explain how different factors are linked to behavior. Commonly used theories in health-related interventions include the Capability, Opportunity, Motivation, Behavior model [14]; Health Belief Model [15]; theory of planned behavior [16]; and transtheoretical model [17].

### Digital Interventions

Interventions to support people with hypertension have the potential to improve outcomes; however, delivery on a wide scale and at low cost is challenging [18]. The use of digital technologies considerably increases the cost-effectiveness of interventions by quickly reaching a large number of people and enabling automation and personalization of content and delivery. Information and Communication Technology solutions such as telephone or video counseling, recorded audio messages, informational websites, and text messages are commonly used in behavioral interventions [19]

A meta-analysis of the effect of text-based interventions concluded that mobile phone text messaging approximately doubles the odds of adequate MA [20]. A systematic review of internet-based interventions for MA similarly found that 11 of 13 studies reported a high or moderate effect on adherence [21].

There is ample evidence that well-designed interventions can improve MA and that digital technologies can facilitate the delivery of these interventions. However, one must navigate a convoluted set of choices to go from theoretical design to practical implementation. A key challenge is that the same theoretical component can be implemented in very different ways, depending on the technology of choice.

### Study Purpose

The purpose of this study was to identify the specific DI components used to target MA or BP control for patients with hypertension and to map those components with respect to their intended change in behavior. Although previous reviews have looked at the effectiveness of DI [22-24] and the behavior change techniques included in interventions [25,26], the specific components of these interventions have not been mapped with respect to their intended effect on behavior and the determinant it is intended to address. We reviewed all studies published in the last decade that describe an intervention with at least one digital communication channel. Then, we identified the digital components and mapped them to the intended behavior and relevant determinant factors using the IM approach [27,28].

This approach allowed us to compare the implementation choices across different studies. The selection of the appropriate theories and components for a given intervention remains unguided. For effective (personalized) DI design in the future, we highlight the most commonly used components and the intended effect of each on patient behavior. This knowledge is essential to evaluate how the specific components have been mapped to behavior change strategies. Furthermore, it supports professionals unfamiliar with behavior change theory in understanding how different functionalities can be used to deliver behavior change components.

## Methods

### Article Identification and Screening

This review intended to answer the following question: “How are DIs designed to improve MA and BP control among patients with hypertension?” We broke this question into three topics: the behaviors, if any, that are targeted for change; the key determinants of the targeted behaviors; and the key components of DIs that effectively improve MA and BP control among patients with hypertension.

We performed a systematic search of the PubMed electronic database. We limited our search to articles published between January 1, 2008, and December 31, 2018, to capture the increasing use of smartphones in health interventions.

### Selection Criteria

In our analysis, we included articles fulfilling all three of the following criteria: articles describing the design and/or evaluation of an intervention to improve BP control and/or MA, studies including the use of digital technologies in the intervention delivery to the patients, and studies with hypertension as an inclusion criterion.

Reviews and surveys were excluded, as were articles written in languages other than English. Studies with sample size smaller than 15 or intervention duration <1 month were also excluded. Since the goal was to analyze interventions, we combined all articles that described the same study.

### Analysis and Categorization

Articles were evaluated by one reviewer and double checked by another reviewer. From each study, we extracted information pertaining to study design (ie, type of study, number of participants, duration of intervention, country), theoretical underpinnings, primary outcome measures regarding MA and BP, intervention delivery modalities, determinant factors, targeted behaviors, and components of the DI. Finally, three investigators in several rounds of discussions verified the results.

In this review, we focused only on the digital components of the intervention to compare how specific digital components were being used across studies. Given the heterogeneity of intervention designs across studies, different theoretical frameworks used, and different technologies employed, we decided to map all studies to a common framework for comparison and for further development of interventions. We selected a format inspired by MoCO as used in the IM framework [13,28].

### Intervention Mapping and Matrix of Change Objectives

IM has been proposed as a 6-step protocol to guide the intervention design process [29]. The second step is to create the MoCO by matching performance objectives (sub-behaviors) with determinants (factors affecting a patient’s conduct). It is important to note that IM can be applied regardless of the underlying theory because it relates to the design process in general.

In IM, matrices that combine performance objectives with their determinants are the basis for intervention development. MoCOs are intended to answer the question “What has to change in a specific determinant in order to bring about the behaviors that need to be changed, to reach performance objectives?” The matrix is created by intersecting the performance objectives with determinants of behavior and environmental conditions.

Performance objectives are statements of what a program participant will do or how an environmental condition will be improved. These performance objectives describe exactly what needs to be done at each environmental level by the at-risk population and the agents or policy makers to achieve health outcome improvements.

Determinants answer the question of “why?” The barriers and facilitators to implementation are considered as determinants. Determinants can be constructed through different methods. They can be theoretical constructs from health promotion theories. They can also be found through comprehensive reviews of empirical literature. Some planners would also investigate qualitative methods used with the targeted population, performed independently or sequentially with a quantitative study using a structured questionnaire with questions based on the results of the qualitative phase, shedding light on more hidden determinants.

Change objectives specify what needs to change in the determinants of behavior or environmental conditions to accomplish the performance objectives.

This framework allows us to organize and categorize existing work in a well-defined structure, offering two benefits. First, such a structure allows for a more in-depth comparison of existing studies in their targeted determinant, behaviors to change, and the components of developed DIs. Second, it facilitates the creation of new DIs, by creating an understanding of the digital components that were previously used, the intended goals, and the areas that have received less or more attention. We created the MoCO by working backwards from each digital component of the DI delivered in each study. We had to identify which behaviors and determinants were addressed, based on the descriptions provided by the authors. After reading all the articles, we needed to understand each intervention component and extract all the DI components first. The second step, which was even more challenging, was to identify the goals behind each component. This rationale was rarely explicitly reported by the author(s) and required interpretation, sometimes requiring extensive discussion, by the reviewers. We identified the addressed determinant(s) and the targeted behavior(s) and then mapped that component to the relevant cell(s) in the matrix. The categories for targeted behavior and determinants were inductively generated from what we identified in the studies we reviewed, with initial inspiration from the Health Belief Model.

## Results

**Figure 1:**
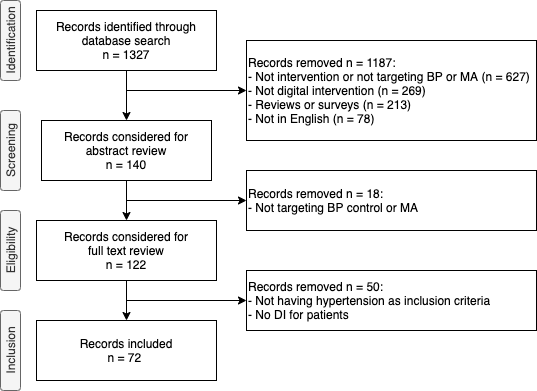
Flowchart of study inclusion. BP: blood pressure; MA: medication adherence; DI: digital intervention.

The literature review resulted in 54 studies that met the research criteria (Figure 1). Because 8 studies published different aspects of their interventions in more than one paper, we mapped all the related articles into a single study for analysis.

### Study Design

The intervention durations ranged from 1 month to 24 months. The most common duration was 12 months (16/54 studies), which seemed more likely to show benefits for improved MA or reduced BP as behavior change occurs over a period of time. Other reported durations were <6 months (11/54 studies), 6 months (11/54 studies), >12 months (7/54 studies), and 7-10 months (7/54 studies). The intervention duration was not explicitly mentioned in 2 of 54 the articles.

Of the 54 studies, 39 were randomized controlled trials, 5 were longitudinal studies, 5 were pilot/feasibility studies, 3 were quasi-experimental studies, and 1 each was an observational study and mixed-method study.

Across the 54 studies, 24 were conducted in North America, 14 in Europe, 8 in Asia, 4 in Latin America, 2 in Africa, and 2 in Australia.

The sample size varied from 19 to 4076 participants. The most common range was 100-499 participants (26/54 studies). The sample size in other studies ranged from 50 to 99 participants (9/54 studies), 500 to 999 participants (7/54 studies), 1000 to 4076 participants (7/54 studies), and 1 to 49 participants (5/54 studies). Almost all the studies had a multidisciplinary team delivering the interventions, ranging from nurses, physicians, pharmacists, social workers/community health workers, and family members.

### Primary Outcome Measures: Medication Adherence and Blood Pressure

Among the 54 reviewed articles, 7 studies focused solely on MA, 18 studies on only BP, and 29 studies on both MA and BP. However, the studies approached those measures in quite different ways, as we explain in the subsequent two sections.

Several papers also included other primary outcome measures, depending on the specific goals of the intervention. Those typically varied between different studies. In brief, they included aspects of body composition (eg, BMI, weight, and waist circumference), scores for quality of life (eg, quality-adjusted life years and quality of life), and the Beliefs about Medicines Questionnaire to demonstrate how their intervention affected patient beliefs about medications. There were 8 studies that performed an economic/cost-effectiveness analysis or resource utilization of the intervention/care costs to demonstrate how important it is to invest more in treatment compliance to avoid emergency room admissions. Self-efficacy was compared using questionnaires in 2 of the 54 studies.

### Measures of Medication Adherence

The papers included in this review used both subjective and objective approaches to determine whether patients took their medication as prescribed. Among the 54 studies reviewed, only 39 reported how MA was measured. In 3 studies, MA was not the primary outcome; instead, it was the secondary outcome [30,31] or used as a measure for medication adjustment [32]. In 8 papers, two separate measures were simultaneously used to determine MA, mainly to reconcile or verify with pharmacy records or pill counts.

Among the 39 studies that reported how MA was measured*,* self-reported values were used in 23 studies. Of these 23 studies, 12 used the Morisky Medication Adherence Scale (MMAS, MMAS-1, MMAS-4, or MMAS-8), 7 studies asked the participant to report whether they had taken the medication, and 1 study each used the 24-item Patient Medication Adherence Questionnaire, Medication Adherence Report Scale, Morisky- Green-Levine Measure of Patient Adherence to Medical Regimens, and Hill-Bone Blood Pressure Therapy Compliance Scale.

Objective MA criteria were used in 24 studies. None of the reviewed studies used direct measures; however, digital medicine (DM) was used in 2 pilot studies. DM is created by attaching an ingestible sensor to each pill that sends a signal to a wearable device when digested. With the help of precise recording of ingestion, the 2 studies were able to capture timing adherence (within ±1 hour around the prescribed dosing time). They were also the only studies that measured ingestion adherence. The most common MA measures were those based on secondary database analyses, such as pharmacy records or dispensing data (11 studies), where 6 studies used proportion of days covered, 1 study used pharmacy refill rate [33], and the remaining 4 studies either did not mention any specific equation or proposed their own. Electronic pill boxes (ePill) or medicine blisters were used in 7 studies, not always as a Medication Events Monitoring System but mainly to count the number of pills/doses taken by the patient. Finally, manual pill count was used in 4 studies.

### Measures of Blood Pressure

BP was the most commonly reported primary outcome measure (47/54 studies). Some studies compared the mean change in BP, either self-measured or measured by a health care professional following specific guidelines, between several time points (eg, every month). Other studies reported the proportion of patients with adequately controlled BP (eg, clinic BP <140/90mmHg). There were a number of studies that only focused on systolic BP since most patients with hypertension who are <65 years old have systolic or a combination of systolic/diastolic hypertension and, for the majority, controlling systolic BP also results in control of diastolic BP [34,35].

To make better treatment decisions, some clinicians prefer to treat patients with hypertension based not only on the clinical measurements but also on additional measurements such as home BP monitoring. There were 21 studies in which patients were provided with home BP monitoring to reduce the white coat effect and allow for multiple and frequent readings, while others measured it during the patient visit following specific guidelines, such as the recommended measurement guidelines from the American Heart Association [36].

### Intervention Results: Blood Pressure and Medication Adherence

We only report the DIs elicited from the reviewed studies. The intervention result is not solely based on DIs, since several of the studies considered a multifaceted intervention that included DI as part of the main intervention. Therefore, the reported intervention results in this section are based on all the components of the reviewed interventions and not only DI components. Since we only focused on BP and MA as the main outcomes, other outcomes are not reported here.

In total, 26 studies reported a significant reduction in BP, whereas 9 studies reported insignificant BP reduction in the DI group.

Regarding MA, 10 studies reported insignificant MA improvement, with 6 studies reporting a better MA rate among patients during the intervention. Another 6 studies reported significant BP reduction and MA improvement, and 2 studies reported insignificant changes in both BP and MA.

There were more studies reporting a significant BP reduction than reporting a significant improvement in MA. This might be because a greater number of studies focused on BP reduction. It might also be due to standardized BP measurements and BP as the final target of MA.

### Theoretical Models and Constructs

Not all studies reported a theoretical underpinning for their DIs; 65% (35/54) of the studies did not explicitly mention the application of psychological theories, models, or principles to their interventions.

Of the 19 remaining studies, the cited theories, models, or principles were behavior change techniques (4/19 studies) targeting patients’ psychological determinants [29], the Chronic Care Model (4/19 studies) to improve care delivery by identifying essential components of the health care system [37], the Social Cognitive Theory (4/19 studies) to change patients’ behavior using their own experiences and observations of others’ actions [38,39], the Health Belief Model [40] (1/19 studies) that is based on patients’ beliefs about the risks and perceptions of the potential benefits of the actions [41], and the Self-Determination Theory (2/19 studies) that highlights the importance of humans' evolved inner resources for personality development and behavioral self-regulation without external influence and interference [42]. Two studies used the principles of patient empowerment as their underpinning cognitive model, and 2 studies used the Common-Sense Model of Self-Regulation [43] that explicates the processes involved in the initiation and maintenance of behaviors for mitigating illness threats.

### Matrix of Change Objectives Components

To map the studies into the MoCO framework, we built the matrix components inductively by reviewing the studies and then mapped them to the corresponding cell in the matrix.

Here, we present an example to illustrate the inductive analysis. The following process was performed for all the studies. All the terms used will be presented further.

Frias et al [44] investigated the effectiveness of DM, in which an ingestible sensor is attached to each medication that sends a signal to a wearable sensor whenever digested. Through the wearable sensor (ie, telemetric devices [TelDev]), physical activity data can also be collected, such as mean daily step count and duration of physical activity and rest. The researchers provided a mobile application for patients and a Web portal for physicians to visualize the DM data: MA, as indicated by ingestion, and physical activity. The mobile device app also prompted the patient to take their medication as scheduled. In brief, the following 3 DI components were used: DM data visualization through the mobile app for patients, DM data visualization through the Web portal for physicians, and medication reminders through the mobile app for patients.

The first component provided visualization of MA adherence and physical activity through a mobile app for patients with the help of TelDev and DM. Without this information, patients were not precisely aware of their medication intake history. Therefore, this component targets the “lack of self-awareness” determinant and “taking medication” behavior through “mobile health (mHealth)” and “DM.” The same interpretation occurs for physical activity through the mobile app and the TelDev; hence, the crossover of the “lack of self-awareness” determinant and “lifestyle management” behavior is addressed through “TelDev” and “mHealth.”

The second component provided DM data visualization to physicians through a Web portal. Thus, this component provides additional information about the patient’s health status and MA. Regarding MA, the DI targets the “clinical inertia” determinant and the patient behavior of “taking medication” through “Web” and “DM.” For physical activity data, the DI focuses on the “lifestyle management” behavior of patients and the same “clinical inertia” determinant through “Web” and “TelDev” at the health care team and system levels.

The last component provides mobile app–based patient reminders to take their medication. Thus, it targets the “forgetfulness” determinant for the patient behavior of “taking medication” through “mHealth.”

### Digital Intervention Delivery Modalities

We extracted all digital components of the interventions that were delivered in all 54 studies. We divided the delivery modalities into the following categories:

- Phone (23 studies): phone calls, including manual (19 studies), Interactive Voice Response (3 studies), and videoconferencing (3 studies)
- Web (26 studies): Web-based platforms
- SMS (13 studies)
- mHealth (16 studies): mHealth smartphone apps
- Email (16 studies)
- Electronic health records (EHR; 7 studies): EHR-based software
- Video (3 studies): non-Web-based educational multimedia content
- Com (2 studies): computer-based programs
- TelDev (8 studies): telemetric devices, including automatic BP monitoring devices or automatic weighing scales
- ePill (6 studies)
- DM (2 studies)

Almost all the studies used a mix of these categories to deliver their interventions. ePill and DM are means of measuring MA. We include them in this categorization for two reasons. First, they are a means of digitalization. With the help of these, one can measure MA without manually entering medication intake. Second, they can be considered an intervention factor, rather than just monitoring, since the act of being observed, especially at such high fidelity, is almost certain to influence a patient’s behavior. Two key factors should generally be considered when discussing MA: monitoring and intervention [45]. Monitoring refers to the means that can reveal whether the patient has taken the medication as prescribed, while intervention refers to the tools that can be used to enhance MA or correct it once a mistake or drift is detected. The use of these electronic devices fulfills both criteria to some extent. The same reasons apply to including TelDev in this categorization.

### Targeted Behaviors

The targeted patient behaviors to change through DI were extracted from 54 studies and then we inductively categorized them into the following:

- PB1: obtain the correct prescription when needed
- PB2: initiate/refill medication
- PB3: take/ingest medication
- PB4: interactive patient-provider communication
- PB5: self-measurement
- PB6: lifestyle management
- PB7: get support from peers or family members

Two-way communication between the patient and provider facilitates resolution of many issues regarding MA and BP control [46,47]. This communication is always happening when the patient visits the provider in person, but that is a relatively rare occurrence. However, through digital communication channels, such as mHealth, the Web, SMS, phone, and email, two-way communication can occur when the patient needs to discuss something with the provider, but in a remote or even offline manner.

### Key Determinants

Although we only included studies that delivered a DI for patients regarding BP control or MA improvement, some studies also simultaneously delivered a DI for the health care team or health care system. Therefore, the key determinants, mainly barriers, were investigated at these 3 levels. Then, we merged the health care team and system levels, resulting in two of the WHO categories: patient level and health care team and system level.

At the health care team and system level, the following determinants were extracted:

- HD1: limited software capability, including a limited appointment system [18,48] and lack of record keeping in EHR [30,49]
- HD2: lack of education, when the health care team had a lack of information about adherence guidelines and general health behavior recommendations or exhibited low adherence to the guidelines [50,51]
- HD3: clinical inertia (aka therapeutic inertia), defined as the failure of health care providers to initiate or intensify therapy according to the current guidelines when treatment goals are unmet [52] (eg, persistently elevated BP), resulting from a combination of health care system, provider, and patient factors [53] including patient factors of low MA [54]. Accordingly, experts recommend MA assessment prior to treatment intensification [55].
- HD4: inadequate health workforce, which occurs when the physician workload is high. Different studies discussed whether to shift some of the workload to pharmacists, community health workers, or nurses or make use of technology.

The latter two were the most targeted determinants.

Regarding the patient level, five determinants were extracted:

- PD1: low perceived risk to health, for which 2 studies aimed to create awareness of the disease risk factors [18,56]
- PD2: lack of education, which occurs when the targeted barrier is a general lack of health literacy or patient/family-based education regarding the different disease conditions
- PD3: lack of self-efficacy, which occurs when the patient barrier is related to a lack of motivation or inefficiency in decision making
- PD4: lack of self-awareness, which occurs when the patients are unaware of their health status recorded either in clinical data (eg, EHR) during visits to doctors or as self-measured data (eg, MA, BP, physical activity)
- PD5: forgetfulness, which occurs when the barrier is multiple medication management or a stressed and busy life, when patients are prone to forget to take their medication

The latter three were the most commonly targeted determinants for behavior change in patients.

### Matrix of Change Objectives

In the resulting MoCO, we listed the delivered DIs in the selected studies with respect to the intended behaviors to change and the targeted key determinant to improve BP control or MA in patients.

Table 1 indicates the matrix for the patient level, and Table 2 illustrates the matrix for the health care team and system level. Of the 54 studies, 63% (34/54) of the studies involved both levels for the DI. We categorized the studies inside each cell based on the delivery modalities.

**Table 1:** Matrix of change objectives at the patient level, with the studies targeting medication adherence or blood pressure improvement in patients with hypertension categorized by digital intervention delivery modality.

Patient behaviour			PD1^a^		PD2^b^		PD3^c^		PD4^d^		PD5^e^
**PB1^f^**											
	EHR^g^		—^h^		[49]		—		—		—
	Email		—		[31]		—		—		—
**PB2^i^**											
	EHR		—		[48,49]		[48]		—		—
	mHealth^j^		—		—		—		—		[57]
	SMS		—		—		—		—		[18]
	Web		—		[48,58]		[48]		—		—
**PB3^k^**											
	Com^l^		[59,60]		—		—		—		—
	DM^m^		—		—		—		—		[44]
	EHR		—		[49,61,62]		—		—		—
	Email		—		—		[63]		—		[31,64]
	ePill^n^		—		—		—		—		[64]
	mHealth		[33,65,66]		[44,61,67,68]		[69]		—		[18,33,57,61,66-71]
	Phone		[49,59-65,72-74]		[75]		[48,58,63,74,76,77]		—		[32,64,72,75,78]
	SMS		[18]		[18]		[18,63,67,79]		[18]		[18,30,50,63,64,67,77,79-81]
	Video		[74,82]		—		[74]		—		[78]
	Web		—		[61,62]		[48,83,84]		—		[61,85]
**PB4^o^**											
	Com		[59,60]		—		[60]		—		—
	EHR		—		[49,86]		[48]		—		—
	Email		—		[87]		[58,63]		—		[84]
	mHealth		[65,69]		[57]		[70,88]		—		—
	Phone		[49,59-65,73,74]		[87]		[48,58,60,63,74,76]		—		[32]
	SMS		—		—		—		—		[18,30,81]
	Web		[51]		[51,60,86,87]		[48,51,56,60,83]		—		[62]
**PB5^p^**											
	EHR		—		[61]		—		—		—
	Email		[60,89]		[31,87]		[60]		—		[31,64,68,71,84,87,88]
	mHealth		[66]		[57,61,66-68,71,86,88,90]		[70,88]		—		[57,68,90]
	Phone		[73]		[72,75,87]		[48]		—		[68,75,87]
	SMS		—		—		[67]		—		[64,67,91,92]
	TelDev^q^		—		[67,68,85,90,93]		[70,85]		—		[68]
	Video		[82]		—		—		—		—
	Web		[31,60,85]		[31,57,60,61,85,87,90,93]		[31,48,58,60,83,90]		[56]		[85]
**PB6^r^**											
	Com		[59,60]		—		—		—		—
	EHR		—		[58,61]		—		—		—
	Email		[60,89]		[93]		[31,58,60,94]		—		[31]
	mHealth		[57,65,66,88,90]		[44,57,61]		[57,61,70,81]		—		[57,61,81]
	Phone		[59-65,72,73,75,95]		[93]		[48,58,76,96]		—		[75,94]
	SMS		[18,67]		[18]		[18,50,79,90]		[18]		[90]
	TelDev		—		[44]		—		—		—
	Web		[31,51,60,85,88]		[61]		[31,48,56,60,61,83,90]		[56]		[61]
**PB7^s^**											
	mHealth		—		—		[61]		—		[68]
	Phone		—		[72,75]		[75]		—		[75]
	Web		[51]		[88]		[51,61,88]		—		—

^a^PD1: patient-level determinant 1, low perceived risk to health.

^b^PD2: patient-level determinant 2, lack of education.

^c^PD3: patient-level determinant 3, lack of self-efficacy.

^d^PD4: patient-level determinant 4, lack of self-awareness.

^e^PD5: patient-level determinant 5, forgetfulness.

^f^PB1: patient behavior 1, obtain the correct prescription when needed.

^g^EHR: electronic health record.

^h^No studies found.

^i^PB2: patient behavior 2, initiate/refill medication.

^j^mHealth: mobile health.

^k^PB3: patient behavior 3, take/ingest medication.

^l^Com: computer-based programs.

^m^DM: digital medicine.

^n^ePill: electronic pill boxes.

^o^PB4: patient behavior 4, interactive patient-provider communication.

^p^PB5: patient behavior 5, self-measurement.

^q^TelDev: telemetric device.

^r^PB6: patient behavior 6, lifestyle management.

^s^PB7: patient behavior 7, get support from peers or family members.

**Table 2:** Matrix of change objectives at the health care team and system level, with the studies targeting medication adherence and blood pressure improvement in patients with hypertension categorized by digital intervention delivery modality.

Patient behaviour		HD1^a^	HD2^b^	HD3^c^	HD4^d^
**PB1^e^**					
	EHR^f^	[49]	—^g^	—	—
	Email	—	—	[31]	—
	mHealth^h^	[30]	—	[30]	[30]
	Web	—	[50]	[70,87]	—
**PB2^i^**					
	EHR	[49]	—	—	—
**PB3^j^**					
	DM^k^	—	—	[44,91]	—
	EHR	[49]	—	[97]	—
	Email	—	—	[64,75]	[63,72]
	ePill^l^	—	—	[64,76,77,97-99]	—
	mHealth	—	—	[67,69]	—
	Phone	—	—	[48,75]	[58,63]
	Web	—	—	[31,44,48,69,70,77,91]	[72]
**PB4^m^**					
	Com^n^	—	—	—	[59]
	EHR	[49]	—	—	[86]
	Email	—	—	—	[63]
	Phone	[49]	—	[70]	[48,58,63]
	SMS	[18]	—	—	—
	Web	[48]	—	[90]	[48,56,86]
**PB5^o^**					
	EHR	—	—	[86]	—
	Email	—	—	[64,67,75]	[72]
	mHealth	—	—	[57,67,88,90]	—
	Phone	—	—	[48,72,75]	[67]
	TelDev^p^	—	—	[64,67,90,91]	[93]
	Web	—	—	[31,48,57,60,70,86,88,90-92]	[72,93]
**PB6^q^**					
	Email	—	—	—	[72]
	mHealth	[30]	—	[29,57]	[30]
	Phone	—	—	[48]	[58]
	TelDev	—	—	[44,91]	—
	Web	—	[50]	[44,48,87,90,91]	[60,72]

^a^HD1: health care team and system-level determinant 1, limited software capabilities.

^b^HD2: health care team and system-level determinant 2, lack of education.

^c^HD3: health care team and system-level determinant 3, clinical inertia (aka therapeutic inertia).

^d^HD4: health care team and system-level determinant 4, inadequate health workforce.

^e^PB1: patient behavior 1, obtain the correct prescription when needed.

^f^EHR: electronic health record.

^g^No studies found.

^h^mHealth: mobile health.

^i^PB2: patient behavior 2, initiate/refill medication.

^j^PB3: patient behavior 3, take/ingest medication.

^k^DM: digital medicine.

^l^ePill: electronic pill boxes.

^m^PB4: patient behavior 4, interactive patient-provider communication.

^n^Com: computer-based programs.

^o^PB5: patient behavior 5, self-measurement.

^p^TelDev: telemetric device.

^q^PB6: patient behavior 6, lifestyle management.

## Discussion

### Principal Findings

To more effectively develop DIs, which leads to more personalized DIs, there is a need to review what has already been developed to identify the gaps. In this paper, we created a MoCO based on 54 reviewed studies to visualize the entire set of previously delivered DIs as a self-explainable map. Moreover, the map can be used to develop future DIs within this domain. With the help of the matrix, we identified a noticeable need to address the intent of the study regarding which determinants were targeted, what behaviors were targeted for change, and which components of DIs have been used. Tailored interventions addressing each patient’s specific barriers to adherence successfully achieve improved MA on a larger scale [100].

Determinants targeted by the studies fell primarily into two levels: patient and health care team and system. This agrees with the results of a systematic review conducted by AlGhurair et al [101] that showed underrepresentation of condition, therapy, and socioeconomic barriers. Thus, there is still room for improvement in DI design by targeting factors from levels other than the patient level. However, according to a review conducted by Klasnja and Pratt [102], health care provider involvement is one of the five key intervention strategies that have been used in phone-based interventions. This also agrees with our inductive analysis, which showed that most of the DIs targeting the patient level (63%) also targeted the health care team and system level.

Using the IM technique can identify appropriate theories for creating DIs. The DIs that did not result in a significant improvement in MA and BP control tended to lack an underlying theory; in fact, 65% of the reviewed studies did not mention the use of theory, models, or principles in their intervention planning, which supports the results from a systematic review of mHealth intervention trials that concluded most interventions failed to incorporate behavioral theories [103]. In contrast, the 10 studies that designed their intervention based on a theoretical model were successful in achieving significant BP reduction or MA improvement or a better rate of BP control or MA.

### Most Targeted Change Objectives

The most commonly targeted change objectives in the matrix were concentrated in the cross between the patient-level determinant of forgetfulness and the taking medication behavior (26/54 studies). One reason for this might be that reminders are one of the easiest and least expensive DIs that can address this change objective. The DIs were mainly delivered through SMS or mHealth, which are considered to attract attention sooner, compared to the Web or email. Moreover, it is less expensive than ePill, is less invasive than DM, and requires less effort from the health care team than phone calls.

The second most commonly targeted change objectives were also patient-level determinants, namely a lack of self-efficacy crossed with lifestyle management (22/54 studies) and a lack of education crossed with lifestyle management (22/54 studies). Lifestyle is an essential element in managing hypertension since optimal therapy involves consideration of the patient’s diet, exercise, tobacco and alcohol use, compliance, and achievement of BP control [104]. The recent guideline for high BP management also provides new treatment recommendations including lifestyle changes as well as BP-lowering medications [105]. Furthermore, dietary risk factors are linked to poor knowledge of hypertension [106]. Evidence has shown that low self-efficacy is usually related to a low level of physical activity [107] and poor MA [101].

Clinical inertia crossed with self-measurement behavior was the third commonly cited change objective (20/54 studies). Clinical inertia crossed with the taking medication behavior was also found in many studies (17/54 studies). Clinical inertia and MA are most definitely intertwined [108], despite being two separate issues in managing hypertension. The results from this review support this fact. Previous studies tried to increase medication intake and self-measurement behaviors of patients to overcome clinical inertia. However, no study focused on the initiation or refill of medication crossed with the clinical inertia determinant. This might be due to several reasons, including patients are provided with medications, patients already have some medications, or the health care team can check medication disbursement via a system (eg, pharmacy pickups). Nevertheless, MA requires the initial purchase or refill of the medication; this could be a focus of future interventions.

### Least Targeted Change Objectives

There were a number of determinants crossed with behaviors to change that were not as commonly addressed by the studies, leading to empty cells in the matrix. If empty cells belong to the health care team and system level, it implies that none of the reviewed studies developed a DI for both levels concurrently. One might find a study that is focused on DIs delivered only to the health care team; however, it was not of interest for this review and was not included. A clear example is many empty cells at the lack of education at the health care team and system level. Only one study [50] had delivered DI to patients and concurrently provided education through a Web portal for the health care team. However, there were some empty cells that were of no interest from a DI point of view. For example, no studies were in the initiation/refill medication behavior crossed with lack of education at the health care team and system level because providing education for the health care team to convince patients to buy or refill their medication does not require a DI or education.

Empty cells at the patient level warrant closer attention since the focus of the review was patient-level DIs. Most empty cells occurred for the get correct prescription when needed behavior. To improve BP control, physicians often adjust antihypertensive medication by increasing the dosage of drugs, switching BP-lowering drugs, or combining different classes of antihypertensive medications [109]. That is why this behavior was targeted at all health care team and system–level DIs but barely at the patient level. Only 2 studies targeted this behavior at the patient level, by increasing patient awareness about elevated BP levels and the need for medication change. However, future DIs can target some of the determinants from the patient level such as a lack of education.

Most of the included DIs did not involve support from family members, especially at the health care team and system level. Assistance and support from peers, family members, and friends can help enhance patients’ optimism and self-esteem, ease the stress of being sick, calm depression caused by the disease, and improve sick-role behaviors, which can improve MA [110]. The limited social support indicates a gap that could be addressed in future personalized DIs.

### Limitations

Systematic reviews, as key elements in evidence-based health care and research, should avoid selection bias. We identified all the relevant studies published from 2008 to 2018 in PubMed. Searching in one electronic database was possibly not the ideal option; however, the number of papers covering DIs delivered to patients with hypertension to improve BP control or MA was extensive. Further, as PubMed is a broad database targeting the area of interest, its use might reduce any selection bias.

To interpret the included articles, the reviewers rechecked and discussed the contents in the framework to gain consensus.

The distinction between the first four patient-level determinants was not always clear. Educational content can contain some information about the risks as well. Since we did not have access to the content of the DIs and the authors did not always describe them in detail, we categorized the DIs according to what was reported in the published articles.

### Conclusions

There is an increasing demand for the wide adoption of digital tools and interventions for patients with hypertension to improve MA and BP control in the short term and quality of life in the long term. To illustrate the analytic results in a self-explainable map based on a common behavior change theory, we built a MoCO using the IM framework. This process highlighted a path for further research in DI design and development in pursuit of MA and BP control in patients with hypertension.

This review highlights the need to design a multi-faceted DI that can be personalized according to patient behavior(s) that need to be changed to overcome the key determinant(s) and considering different levels including patient and provider involvement.

## Abbreviations

BP: blood pressure
DI: digital intervention
DM: digital medicine
EHR: electronic health record
ePill: electronic pill boxes
IM: Intervention Mapping
MA: medication adherence
mHealth: mobile health
MoCO: matrix of change objectives
TelDev: telemetric devices
WHO: World Health Organization
